# Extracts and Gleanings

**Published:** 1873-04

**Authors:** 


### EXTRACTS AND GLEANINGS FROM OUR EXCHANGES.

### *MULTUM IN PARVO.*

An Old Remedy in a New Role.—I wish to call the attention of the profession to an old remedy in new *rolé*. For one year I have been using poultices, made in a decoction of the leaves of digitalis, to all swellings or enlargements where there was any prospect of the formation of an abscess or the secretion of pus. It has succeeded better than anything used, where an abscess was threatened in the breasts of nursing women.

I could give the history of a number of cases, but will forbear, desiring to be as brief as possible, to be understood.

For an ordinary-sized poultice: ℞. Fol. digital., ℥jss.; aqua bul., ℥xviii. Boil briskly thirty minutes, and stir in Indian meal or, what is better, ground flax-seed, to a proper consistence. Apply warm. Repeat as often as it becomes dry and the cause for using exists.

A. G. Smythe, M.D.

Near Gun Town, Mississippi, March 10, 1873.

A Substitute for Quinine.—I see in the number for February of your journal a short article on the use of carbazotate of ammonia as a substitute for quinine.

I have used the muriate of ammonia in a number of cases where the use of quinine had become disagreeable in recurring intermittents, with the happiest effect.

Fifteen grains in a little water every two hours for eight or ten hours before the approach of the chill. Repeat for several days.

A. G. Smythe, M.D.

Near Gun Town, Mississippi, March 22, 1873.

Vascular Cornea-Pannus—Granulated Lids.—The local application of quinine ℈j., morph. sulph. grs. iij., rubbed well together in water till thoroughly mixed, and a portion applied *dry* by means of a fine brush upon the cornea and on the lids, has been very successful in removing these affections.—*The Doctor*.

HOOPING-COUGH.—Dr. Thomas D. Davis, (*Philadelphia Medical Times*), reporting a trial of chestnut leaves in hooping-cough, writes: The first eleven cases all had the accustomed hoop; the remainder had well-marked paroxysms, but not the full spasm, and they recovered without having it. In each case the violence of the spasm was reduced even more markedly than the number of the paroxysms. On the days on which the cases are marked as having no paroxysms, they had slight coughs, but no sign of spasm whatever. The castanea was continued for a week, after which, in a few cases, a simple expectorant was given. The nurse in charge, who had witnessed many epidemics of the disease, declared she had never seen a medicine act like it.

This medicine is made from the leaves of the common chestnut tree—Castanea vesca, natural order Cupuliferæ. Another species of this genus, Castanea pumlia (chincapin), for some time held a place in the secondary list of our Pharmacopœia; the bark of this was an astringent and tonic, and was employed also in the cure of intermittents. The first mention I can find of chestnut leaves as a remedy was in a paper read before the American Pharmaceutical Association, in 1862, by Mr. George C. Close, of Brooklyn, New York, (*American Journal of Pharmacy*, 1863, p. 66). They were recommended to him as a cure for pertussis by a "prominent physician of New York," whose success with them had been very marked. He administered an infusion of them made with boiling water and sweetened with sugar, and reports that in every case it "acted like a charm."

Dr. J. Unzicker, of Cincinnati, called attention to this remedy, with a very favorable account of its virtues, in an article written in 1867, (*Medical and Surgical Reporter*, October 26, 1867, p. 355). He claimed that it had never failed in his hands to cure or relieve the spasm and bring the disease to a speedy end.

Dr. J. Ludlow, of the same city, commended it highly in articles written two years later, (*Cincinnati Lancet and Observer*, March, 1869, p. 147, and *New York Medical Journal*, April, 1869). He gives the credit to Dr. Unzicker for having first brought it to his notice, and says: "In every case that I have treated since, I have invariably used it, and with the happiest result—in from five to ten days relieving the spasm, and in about two weeks curing it." All of these observers used an infusion of the leaves.

In a paper presented to the Philadelphia College of Pharmacy, November 21, 1871, by Mr. John M. Maisch, (*American Journal of Pharmacy*, December, 1871, p. 529), he announces that since 1867, at the request of Dr. A. S. Gerhard, of Philadelphia, he had collected chestnut leaves, and had prepared of them an infusion, syrup, and fluid extract, of which he greatly preferred the latter preparation. He speaks in the highest praise of the medicine in hooping-cough, but mentions two cases in which it did not appear to be of benefit. Mr. Maisch is the only druggist who has preparations of this article; it was his fluid extract that we used in the cases reported. The leaves are gathered from July to October, the preference being given to those procured late in the season. The following is Mr. Maisch's formula for making the fluid extract: Chestnut leaves, dried, cut, and bruised, sixteen ounces; glycerin, five ounces; sugar, eight ounces; and hot water, a sufficient quantity; the extract to measure sixteen fluid ounces. His method of preparing this extract is given in his paper. The dose is half a teaspoonful to a teaspoonful every three or four hours, for a child six years old. In urging this remedy upon the notice of the profession, we do not claim for it a specific action, but think it will be found a valuable anti-spasmodic and expectorant, the preparations of which are not unpleasant, and are, therefore, readily taken by children. In the cases we have cited, we are sensible that in the most of them treatment was begun in the hight of the disease, and we might consequently expect a decrease in its severity, but scarcely such a marked decline as our cases indicated under the use of the chestnut leaves. As a domestic remedy, an infusion of the leaves has been used for many years in regions of the country where this tree flourished.

Arsenic in Cutaneous Diseases.—Dr. Anderson McCall says:

"1. Arsenic, judiciously administered, is as safe as any in the Pharmacopœia, and may often be used for months without injury to the general health.

"2. It often requires to be continued for many weeks, and sometimes the disease seems to resist its action for a considerable time, when all of a sudden improvement occurs, followed by a rapid cure.

"3. It requires to be given in proportionately larger doses to children than to adults.

"4. Infants may be subjected to its influence by administering it to their nurses.

"5. The dose should be at first small, and not increased, as a rule, for some time. Then it may be gradually increased till the medicine disagrees, or till the disease begins to yield, when it may as gradually be diminished.

"6. It should not be omitted altogether without very good reason, but may be tried in smaller doses or in another form, or omitted for a few days, till the bad effects have passed off.

"7. Puffiness of the face, or irritation of the eyes, or such like physiological effects, if slight in degree, should not lead us to discontinue the medicine; indeed, it is sometimes only then that its beneficial action on the disease is observed.

"8. It is decidedly contra-indicated in acute diseases, and when its use is followed by marked increase of irritation of the skin—itching, heat, &c.—the disease is probably not in a state to be benefited by it.

"9. It is generally more rapidly effectual if the disease, though in a chronic state, is recent; and the first attacks yield more readily to it than subsequent ones, as a rule.

"10. It is contra-indicated in most cases which are complicated with digestive derangement.

"11. It is apt to produce bronchial catarrh, so that patients should be warned to avoid exposure to cold while taking it; and for this reason it is generally contra-indicated in persons laboring under bronchitis.

"12. In exceptional cases it may be given with benefit in large doses.

"13. It sometimes requires to be given during meals, or immediately after food is taken, for when administered on an empty stomach, it occasionally deranges the digestive organs; and it is often better tolerated if given along with a bitter infusion.

"14. It should not, as a rule, be entirely discontinued until some weeks have elapsed since the complete disappearance of the eruption.

"15. There are few chronic diseases of the skin of constitutional origin—provided they are not syphilitic—which may not be benefited by it (although often other treatment is to be preferred to it), but it is especially valuable in psoriasis, pemphigus, lichen ruber, pityriasis rubra, and in many cases of eczema, unless contra-indicated as above."

On the Internal Use of Carbolic Acid.—*By James Allan, M.B., L.R.C.S.E., Newmilns, Ayrshire.*—In this paper Dr. Allan points out shortly diseases in which he believes, from the results he has obtained, carbolic acid may be used with success. In the treatment of mucous tubercles and primary syphilitic sores, he has met with a considerable amount of success. The method of using it is by applying a solution of equal parts of carbolic acid and water once daily, and by the constant application of a lotion of eight grains of carbolic acid to the ounce of water. If the acid be applied in a concentrated form, it causes the sore to assume a whitish appearance, after which a thin yellow scab forms, which separates in three or four days. In all cases the pain was inconsiderable, and the destruction of the sores rapid. According to the trials in which Dr. Allan has employed carbolic acid in the treatment of syphilis, he is unable to form a definite opinion as to its merits or defects. In two cases which resisted all remedial means it was prescribed, and in a few weeks all the symptoms had disappeared. The effects have also been investigated by Dr. Köhn, in Hebra's wards, in whose experience the beneficial results were not so well marked as to recommend its administration.

Dr. Allan agrees with many observers as to the beneficial effects of carbolic acid in the fermentive class of dyspeptic cases, in which there is flatulence with evolution of gas, with a tendency to vomit. For this it can be administered either in solution in grain doses, or in the form of a pill. Thus given, it stimulates the stomach, checks the fermentative process, and reduces an evolution of gas and evacuation of flatus. In hemorrhagic ulcer of the stomach, a few administrations of grain doses, freely diluted, are very efficacious in checking the bleeding, provided, as in all cases, due attention has been paid to the diet. Good results have also been obtained in cases of chronic gastric catarrh, especially if some stomachic sedative be first given. It should be given upon an empty stomach, in quarter to half-grain doses much diluted.

In considering the different processes which take place during the decomposition of the pulmonary tissue in phthisis, on account of the deteriorated state of vitality, it would seem that, if an antiseptic could be introduced by any means which would not derange the general functions, the disease might be arrested. From a careful review of the cases recorded in the periodical literature, as well as from results of practice which Dr. Allan has himself seen, he is certain that when pulmonary disorganization has taken place, carbolic acid has no effect to eradicate it. In the treatment of certain skin diseases, Dr. Allan believes carbolic acid to be of much use, especially in those of an obstinate nature. Good results have been obtained in psoriasis, pityriasis, and prurigo. It should be administered in the form of pills, each containing one grain, and from six to nine of these given daily, and gradually increased. In gangrene of the lungs, it removes the fetid odor, and seemingly produces good. In chronic bronchitis and oozing hemorrhages of the air passages, it is useful; so, also, in mucous diarrhœa of the large bowel, in the form of an injection, when preceded by some alkaline solution.—*British Medical Journal.*

Treatment of Gonorrhœa.—Mr. McDonald recommands the following: Smear a bougie with ointment of the nitrate of silver, (℞. Argent. nitratis, ʒj.; adipis, ℥j.;) introduce it into the urethra about three inches, and allow it to remain two or three minutes. Two or three applications have been found to cure the disease.

Black Cohosh.—A writer on this article says: "I have repeatedly seen it used as a substitute for the ergot, and in every case with the most happy effect. It has the desired properties as a partus accelerator, without the power of doing injury to the fœtus, as sometimes does the ergot. Also it does not diminish the susceptibility of the uterus to succeeding doses of the medicine, as is the fact when the ergot is given. In simple, large and full doses, it produces convulsive contractions of the muscles of involuntary motion, and first of the uterus. Thus it is narcotic, and ecbolic because of its peculiar narcotic operation. When given in inordinate quantities, it will produce convulsive action of the heart, and painful palpitations. Besides being narcotic, it operates in a peculiar manner on the glandular system. Thus it increases or diminishes the secretion from the liver, when its secretions are morbidly diminished or increased. It removes topical inflammations of the atonic kind, without an evacuation. It affects the bronchial mucous membrane, diminishing morbidly increased expectoration. Diaphoresis and diuresis often follow the use of the article. It has an action on the cutaneous surface, differing from a diaphoretic, producing resolution of the diseases peculiar to the part. It is emenagogue. It removes pain of the neuralgic kind, thus being a valuable medicine in colic. On some patients it has a cathartic operation, though this is but rarely noticed.

"For the removal of rheumatism, whether acute or chronic, it is the Samson of the Materia Medica. The gates of Gaza, posts and all, are sure to start, if it be properly given. It is necessary, in order that the medicine may operate as we wish in this or any disease, that the strength of action in the circulating system be as much as possible short of phlogistic diathesis, and any reduction below this point by bleeding or purging, will be injurious. It should be given every hour, and continued till some symptoms of ultimate narcosis appear—then hold on for a few hours and resume again, if necessary.

"In ophthalmitis conjunctiva, its effects will be as distinctly seen as in any disease. A short time since I was called to administer to a maiden lady suffering with this affection. The prescription was tincture actæ racemosa, one drachm every hour. As some topical application was, of course, expected, and none being prescribed, the gentleman of the house sportingly inquired why I did not extract a tooth to cure the eyes? In a few hours the intolerance to light was mostly removed, and the second ounce of the medicine completely cured the disease. In other cases it has had the same immediate and happy effect, no other medicine being given. It is said that it has been given to gravid females for the removal of cough, with the effect of producing speedy abortion.

"The best possible manner of giving it is in the form of tincture, which should be made with four ounces of the root, finely powdered, to one pint of undiluted alcohol. If the alcohol be diluted, the value of the tincture will be much deteriorated. The watery infusion is of no account. Given in fine powder it is not inert, but it is not the best preparation. Alcohol seems to be its proper menstruum, and the stronger the alcohol the better."—*Eclectic Medical Journal.*

THE SCIENCE AND ART OF HEALING WOUNDS.*—*By Benjamin W. Richardson, M.D., F.R.S.*—After alluding to the great diversity of opinion existing in the profession on the subject of treating wounded surfaces, Dr. Richardson illustrated from clinical observation the results of healing, in extreme cases, by the first intention, and then he put the inquiry—Why, if in one case of the extreme kind named there could be cure by the first intention, there should not be such cure in the majority of cases—why, in short, should success by this method of cure be the exception instead of the rule? Two sets of causes stood in the way—one set remediable, the other irremediable. The remediable obstacles to recovery by the first intention were—want of care in bringing divided surfaces into perfect apposition; the too free use of water in dressing; the too prolonged exposure of wounds to the air; too much manipulation of the surface of the wound; the leaving of long ligatures within the wound; the imperfect closure of wounds from the air; the too hasty removal of dressings; and, lastly and most important of all, error of judgment on the part of the dresser in respect to the question whether there be, in the case to be treated, sufficient continuity of surface to warrant the attempt to heal by the first intention. The irremediable causes preventing healing by the first intention were—nervous lesions, influencing the vascular supply of the injured part; the accidental introduction into the wound of decomposing or other foreign matter, or the generation of organic poisonous product within the body. These cases lie apart from the science and art of dressing wounds, and may be left for a special and future discussion. For the promotion of healing by the first intention, the author contended that the dressing employed should have four distinct qualities: it should be collodial, elastic, impermeable to air, and styptic.

Healing by the second intention formed the next part of Dr. Richardson's communication. Here again the rules named in relation to healing by the first intention were considered, and it was maintained that the same collodial dressing was as effective in

*Read at a Meeting of the Medical Society of London, January 29.

curing by the secondary as by the primary process, the difference of application being that the whole of the exposed surface was to be treated with the collodial fluid. After giving a series of cases of rapid healing by the second intention, cases in some of which bone had been exposed, and even a joint laid open, and after touching briefly on the addition of iodine to collodial solution in some particular cases, the author placed before the Society a summary of his argumtnt in a series of short propositions, and concluded by insisting that medical men live to cure all they can, and to try to cure everything until they succeed, through whatever tribulation they may pass in the labor.—*Lancet.*

COLOTOMY FOR INTESTINAL OBSTRUCTION (*Medical Times and Gazette*, August 24, 1872).—Mr. Steele commences by observing that many cases of intestinal obstruction terminate fatally without surgical interference, which, were timely operative measures adopted, would very probably end in recovery. He relates the case of a man, aged fifty-two, who, usually enjoying good health, had lately suffered from diarrhœa. On June 2d he was unable to relieve his bowels; he took castor oil, but without effect. Mr. Steele saw him early next day, and found tympanites, colicky pains, and fæcal accumulation in the rectum, with strong desire for defecation. Various aperients and enemata were unavailing; the rectum was cleared out, and galvanism was applied, but without result. Bad symptoms soon set in, succeeded by failing power of the heart. This was relieved by ether and laudanum. Liquid food was well taken and retained. On the sixth day, the patient, who had somewhat rallied, suddenly becoming worse, colotomy was performed. Flatus immediately escaped, and fæces some few hours afterwards. Localized peritonitis, inflammation of the skin, diarrhœa, gastric and intestinal irritation, etc., gave great anxiety for about four weeks. By this time the wound was well healed round the intestine, and the patient improved, and became restored to fair health, but remained weak. No passage per rectum had since occurred, but free discharges of thick mucus had proved troublesome. A swelling high up in the pelvis, which before operation seemed like fæces accumulated in the intestinal coils, afterwards descended, and proved to be a tumor, and the cause of obstruction. The patient was doing well. Mr. Steele concludes with observing that where the cause of obstruction is obscure, and appears to be fæcal accumulation, all legitimate endeavors should be made to dislodge the same; that when the cause of obstruction is clearly mechanical, opiate treatment should be at once commenced, and operative interference promptly adopted; that in such a case as the one narrated, surgical aid is the only means of saving life; that a person with a tumor compressing the lower bowel is in a much better condition with an artificial anus than with a constantly forced passage by the natural orifice; that the growth of the tumor will not be nearly so rapid as if it were subject to compression by the fæces and strained defecation; and that operation is most likely to be successful when the obstruction is caused by tumor, there not being sloughing to fear, as in internal hernia or intussusception.—*Philadelphia Medical Times.*

Treatment of some Affections of the Nasal Cavities. In the course of the winter, various forms of disease occur in the northern regions of Italy, which have their starting point from, and their seat in, the nose. For erythema, a troublesome and painful accompaniment of acute ozœna, attacking the upper lip, it is customary there to apply, three or four times a day, glycerized starch paste twenty parts, laudanum one part. This affection, when once excited in infants at the breast, is very persistent, and the above remedy answers well. Demarquay recommended the injection into the nostrils, by means of a glass syringe, of a little glycerin and water. In cases where the coryza is of a chronic character, the following prescription is stated to be very successful in effecting a cure: Aq. rosarum, sixty parts; glycerin, sixty parts; tannin, one part. The use of glycerin is well adapted for cases accompanied by a disagreeable smell in the nose, and the frequency of the application should vary with the intensity of the affection. If the suppuration be of a syphilitic nature, the glycerin may be combined with calomel, or with binoxide of mercury. In scrofula, a little iodine may be added to the glycerin. The ill odors accompanying ozœna may also be almost infallibly removed by means of injections of solutions containing the permanganate of potash in the proportion of one part of the salt to ten of water. Darcey injects into the nostrils a solution of 0.12 of tannin, 1.75 of glycerin, and 2.80 of water. Galligo, on the other hand, prefers injecting a solution of eight parts of chlorate of potash in 100 of glycerin. Glycerin and starch readily lend themselves to the application of mercurial, lead and iron salts; and glycerin is useful in cases of odorless nasal humors, and herpes of the nose and lip.—*Gazetta Medica Ital. and Aerztiches Literaturblatt.*

Hemorrhage from the Nose.—Introduce the little finger into the nostril, and press upon its floor until the bleeding stops; then take a dossil of lint, and roll it upon powdered alum, and press it upon the floor of the nostril with the little finger. Introduce pieces of lint, in this way, until the roof of the nostril supplies the pressure of the finger.—*O.*

Conservative Surgery in Minor Operations.—Dr. A. P. Grinnell says, in the New York *Medical Journal:* A lad, aged fourteen, of a vigorous, healthy constitution, received a severe wound on the right hand by a circular saw. Upon examination of the injury, I found that the saw had entirely severed the third finger, with the exception of a little cuticle, which held the end suspended between the first and second articulation, had disarticulated the first and second phalanges at the second joint, and, besides, injured the soft parts in different parts of the hand. My first thought was to finish the amputation of all dependent portions, but, upon reflection, I determined to afford nature an opportunity of showing her skill in establishing a union of these lacerated tissues. The subsequent treatment involved much care and attention, but the result was sufficiently gratifying to reward the effort of preserving the whole hand. We have been taught that, in all injuries of this character, where union has taken place, sensation was lost, and the power of motion considerably impaired; but, in the above case, bone united firmly to bone, tendon with tendon, nerve with nerve, and the circulation of blood was fully restored. Sensation is perfect beyond the point of injury; and, to have secured this result, nerve must have been united with nerve, for the amputations of all sensitive portions was complete. The power of motion is also preserved, although not perfect in one finger, owing to anchylosis of a joint.

Nutritive Enemata.—It has been maintained by the physiologists, Steinhauser and Beclard (*Gazette Hebdomadaire,* August 23, 1872), that the capability of digestion of the large intestine, which in its normal state is exceedingly small, may become more active when the juices of the small intestine, not being employed in the process of digestion, flow into the cœcum. M. Leube suggests that this discovery should be utilized for the benefit of invalids, and he therefore alleges the possibility of supporting life by throwing into the large intestine certain digestible substances mixed with a digesting agent. The latter consists of the pancreas of swine.

The enema which he suggests is a hash consisting of fifty to one hundred grammes of the pancreas of a cow, freed from all fat, and one hundred and fifty to three hundred grammes of beef. These two substances are pounded up in a mortar, and suspended in a sufficient quantity of warm water. The injection of this compound, it is asserted, is never followed by diarrhœa. On the contrary, it is generally retained in the intestine for a period varying from twelve to thirty-six hours. For the digestion of albuminous substances, it is thought that pepsin might be more efficacious.—*Boston Medical and Surgical Journal.*

ANTISEPTIC INJECTIONS AND DRAINAGE TUBES IN EMPYEMA. —*By Edmund Andrews, M.D., Professor of Surgery in Chicago Medical College.*—The great success of carbolic acid injections in arresting the purulent secretion, and consequent exhaustion and hectic of large abscesses, determined me to try it in empyema. The following case shows the success:

Michael Hennessy was stabbed in the back part of the left chest last June. The wound was closed, but effusion occurred, which became purulent, and discharged through a fistulous orifice, remaining after the tapping of the cavity. A few months later he was found confined to the bed, emaciated, and slowly going down with the purulent suppuration.

My first effort was to inject carbolated water into the fistula, but I found the opening small and crooked, and the interior so full of pus that not enough of the antiseptic could be gotten in to accomplish anything. I then anæsthetized the patient and made a good sized opening into the chest between the ribs where the fistula lay. About a quart of pus gushed out, showing that the contraction of the orifice had kept in the discharge, and prevented the tendency of the cavity to gradually collapse and become obliterated. To prevent a recurrence of the trouble, I inserted a small rubber tube, and tied it in. Through this tube large injections of carbolated water, ten grains to the ounce, were freely thrown once a day. Between the dressings, the wound and the orifice of the tubes were kept guarded by a compress of cotton dipped in carbolated oil, forty grains to the ounce. By this treatment the secretion of pus has been almost arrested; the patient is growing fat and vigorous, and is already able to take long walks. The cavity is becoming smaller, and bids fair to be safely obliterated.

Experiments made by Dr. Andrews, in Mercy Hospital, show that although cundurango has no control over cancerous tumors, it powerfully promotes the growth of granulations in chronic indolent ulcers. He promises shortly to give us his facts in full.—*Chicago Medical Examiner.*

GENDRET'S BLISTERING OINTMENT.—Simple cerate, ℥i.; oil of sweet lavender, ℥ss.; melt these together, and then pour them into a wide mouth glass vessel; then add ℥v. of strong aqua ammonia, stirring the mixture all the time. The melted mixture must not be too warm, otherwise a portion of the ammonia will escape. The ointment thus prepared retains its properties for months, if kept cool and in well-stopped bottles. It produces vesication in about ten minutes, and is applied by spreading on the skin and covering the part with a compress. The manner of preparing it is simple, and the manner of application ready.

On Vaccination and Revaccination of Pregnant Women.—The question has frequently been put to Dr. Barnes, Is it right to vaccinate pregnant women? Some persons seem to entertain the apprehension that pregnant women incur special and serious risks under vaccination. To justify exceptional neglect of vaccination in their case, it ought to be shown, not only what this special risk is, but also that it is more serious than the risk incurred by the women themselves by taking the small pox, and thus of propagating the disease to others. The community, as well as the pregnant woman, must be considered.

To make out, then, a case for special exemption, it ought to be shown that the pregnant woman incurs a peculiar danger. Where is the evidence of this? Dr. Meigs says: "Do not vaccinate women when pregnant. I have been the witness of dreadful distress from the operation. Eschew it, I entreat you." Dr. Barnes fears there is some confusion in the matter. His own experience has supplied him with many illustrations, which warrant the following propositions:

1. Pregnant women living under epidemic or zymotic influences are more prone to take the prevalent morbid poison than others.

2. Having taken a morbid poison, they are less able to throw it off.

3. Their system is less able to resist its injurious action. Abortion and a most dangerous form of puerperal fever are very likely to follow.

Dr. Barnes thinks we may conclude, in the absence of decisive evidence of special danger, that pregnant women are entitled to equal protection against small pox with the rest of the community; and that vaccination or revaccination should be practiced on pregnant women, in their own interest, as well as that of the community of which they form a part.—*British Medical Journal.*

Cimicifuga in Enlargement of Spleen.—A writer in the *Eclectic Medical Journal* says: From its almost specific action on the spleen, I was led several years ago to use it in intermittent fever, since which I have used it for this disease, with invariable success, in the following form: Put fifteen grains sulphate quinine into a two-ounce vial, add elixir vitriol just sufficient to dissolve the quinine, and fill the vial with the saturated tincture of actæ; of this, an adult must take twenty drops every hour during the intermission. Black cohosh root has been highly recommended as useful in arresting hemorrhages of all kinds, for coughs, bowel complaints, as a gargle for the quinsy, etc. Its most active properties reside in its resin. It may be used in powder, saturated tincture, resin, or hydro-alcoholic extract; the decoction is an indifferent preparation.

Sulphurous Acid Lotion in the Treatment of Contused Wounds.—Dr. John Balfour states that an extended experience has given him great faith in this application. It gives almost instant relief from pain, controls and greatly restrains suppurative action, and, where possible, secures primary union perhaps as efficiently as carbolic acid. The lotion is of the strength of one in twelve; a thin rag (the thinner the better) should be laid over the wound, and kept constantly wet, for the first thirty-six to forty-eight hours. When cold becomes less agreeable, the lotion is used tepid, the rag being wetted every twelve hours and covered with gutta-percha. Where primary union is taking place, about the third or fourth day, a dressing of zinc ointment is to be substituted for the washing; this allows the skin to heal. When suppuration is established, a zinc lotion may be used after a week or ten days, and the cure wrought out on ordinary principles. Dr. Balfour records the following, amongst other cases: S. B., a lad between eleven and twelve years of age, on the 8th of June, in company with some other boys, was amusing himself with gunpowder; a "peeoge" (or devil) hung fire, and he poured some powder on it from the flask. This, of course, exploded, and tore open the metacarpal space between the thumb and forefinger of the right hand. The metacarpal bone of the thumb was fractured, and both wrists scorched. A mass of the short flexors of the thumb was forced out of the wound, contused, torn and blackened. As this muscular substance was much injured and could not be returned without using undue force, a good deal of it was cut off; the wound was washed out with the sulphurous acid lotion, covered with a wet rag wet with the same, and the fracture was kept in position by tying the thumb to the forefinger. Had a fair night's rest; the wrists (not complained of yesterday) now painful and beginning to vesicate; dressed with carbolic acid and oil. Everything went on well; the burns on the wrists healed kindly; suppuration was most moderate; cicatrization rapid and perfect. Dr. Balfour lately passed the boy into a public work, with a thumb very little, if at all, the worse for the accident.—*Edinburgh Medical Journal.*

Treatment of Irritability of the Stomach.—℞. Strychnia, gr. j.; acidi nitrici, dil., ʒi.; aquæ, ℥xij.; solve, at sumat æger, fiat ℥j. *ter in die,* and rub the scrob. with a liniment of croton oil; milk dietary, consisting of eighteen ounces of bread, one ounce of butter, and two pints of milk daily. The medicine to be taken fifteen minutes after each meal. The strychnia acts particularly upon the spinal marrow, and it is supposed that when alkaline urine is secreted, independently of the character of the ingesta, there is always some lesion of this part.

The Fever Tree.—Dr. Pepro L. N. Chernovis, of Bahia, in a late number of the *Gazeta Medica la Bahia*, gives a very interesting account of the history, uses, propagation, medical and miscellaneous properties of the *Eucalyptus Globulus*, an immense tree introduced into various provinces of Brazil from Australia, and called, as in Spain, *arvore de febre*, from its "marvelous results in the treatment of intermittent fevers." The tree is colossal, sometimes attaining a hight of three hundred feet, and a diameter of thirty feet; ranging often from fifty to seventy feet in hight, and ten to twenty feet in diameter. All parts are aromatic, less so in the trunk and bark, more so in the small roots, flowers and leaves. It is a comparatively new medicine of much promise, and is given internally for intermittent fever in doses of from one to four drachms of the powdered leaves—twice during the intermissions—or in infusion (two drachms in four ounces of boiling water) morning and evening. Aqueous and alcoholic extracts, in doses of from two to eight grains, are also used for the same disease. One or two drops of its essential oil, on sugar, in pill or capsule, are advised in bronchial and pulmonary affections, laryngitis and catarrhal aphonia.

Carbazotate of Ammonia in the Treatment of Malarial Poisoning.—Dr. Beaumetz brought, in a recent paper before the Société de Thérapeutiqe de Paris, the above named substance to the notice of the profession. He related the successful employment of this salt by Braconnot, Calvert, Aspland, Bell, Manopa, etc., and gave the results of six cases treated by himself, of various experiments carried on upon animals and man. Like quinine, it diminishes the strength of the pulse, brings on heaviness, cephalagia, and even delirium, and is eliminated by the kidneys. From his experiments and cases, Dr. Beaumetz draws the following conclusions: Carbazotate of ammonia is very efficacious in intermittent fever; the suppression of the paroxysms may be obtained by the use of one-third to two-thirds of a grain daily, given in three doses; the drug never has any bad effect, and seems to be better tolerated than sulphate of quinine, though its physiological action is very similar.—*London Lancet.*

Treatment of Hemorrhage from the Bowels in Typhoid Fever.—Dr. S. Weed (*Buffalo Medical and Surgical Journal*) reports five cases in which this complication arose, in some of them the loss of blood being very great. All the cases recovered under the use of oil of turpentine. It was given at first in teaspoonful doses once an hour, or oftener if necessary, and after two or three repetitions, twenty drops were prescribed to be taken every two hours.

On Thoracentesis.—Dr. Clifford Albutt (*The Practitioner*) urges early puncture of the thorax in cases of pleuritic effusion. He thinks that nothing approaching the *promptitude* of the relief which in such cases is afforded by thoracentesis can be attained by medicine. He states that if he finds any large amount of fluid in the pleura of a patient, he now never tries medicine at all, unless the friends or the family attendant urge such a course; for in his records of cases treated by drugs, and in such records as he has of cases so treated by others, the results are very far inferior to those in which puncture has been employed. He cites the case of an old gentleman who had gone about for many months with his left pleura nearly full of fluid, and who had been treated with mercurials, digitalis, iodides, tonics, and good nutrients, but in vain. In the end, the fluid subsided in considerable measure, but chronic pneumonia supervened in the injured lung, and he ultimately died. "I have no reasonable doubt," he states, "that his life could have been spared, and his health quickly restored, had he been tapped at the beginning."

Lobelia in Small Doses.—Tincture of lobelia, (sem.) in doses short of nausea, is one of my favorites. If you have disease of any mucous membrane, with tendency to congestion, it is a direct remedy. I have used it this fall with marked success, in diseases of the respiratory mucous membranes, analogous to hay-fever. The prescription was: ℞. Tinct. aconite, tinct. lobelia, (sem.) aa. gtts. v.; water, ℥iv. A teaspoonful every one, two or three hours.

*Asthenic Lyringitis and Pneumonia in Childhood.*—Talking of lobelia brings to mind that it is *the* remedy in asthenic disease of the respiratory apparatus in childhood. My old prescription was: ℞. Tinct. lobelia, ℨj.; comp. spts. lavender, ℨiij; simple syrup, ℥jss. M. Half a teaspoonful every one, two or three hours.

Now I prescribe it from my pocket case: ℞. Tinct. lobelia, (sem.) gtts v. to x.; Tinct. aconite, gtts. ij. to v.; water, ℥iv. A teaspoonful every hour.—*Dr. J. M. Scudder, in Eclectic Medical Journal.*

Ichthyosis Cured by Sulphate of Copper.—Dr. Dumesnit reports a case of ichthyosis of the nose, which had resisted various remedies, internal and external. Dr. D. then prescribed an ointment of four parts of sulphate of copper in thirty parts of benzoated lard, under the use of which the cutaneous affection disappeared in about three weeks. It returned, however, a month later, when the same application was resumed and persisted in, and the patient continued free from the disease up to the date of the report, two years after the remedy was first used.—*Report de Pharmacie.*

Strumous Ophthalmia in Children.—Von Graefe recommended that the face of the child be dipped in cold water once a day till the photophobia and other symptoms subsided. The following is his method: Two assistants take charge of the child—one secures the lower extremities, the other takes charge of the body and upper limbs; then the surgeon seizes the child's head and dips its face in a vessel of cold water, and retains it there for ten seconds, and repeats a second time. He describes the effect as magnificent on the blepharospasm. Then a solution of atropine, one grain to the ounce of distilled water, is carefully distilled in the eye. The application of extract of belladonna ʒj., to glycerin ℥i., to the outside of the lids or the temple and over the eyebrows, gives a great deal of relief. This is to be applied night and morning. Glycerin ʒss., internally administered in water, three times a day, and the dose gradually increased to ʒj., has done good service in curing these cases. An infusion of the root of the *hydrastis canadensis* makes a very good collyrium in simple ophthalmia of children. The decoction applied to ringworms cure them.—*Medical Archives.*

Syrupus Cubebæ.—A correspondent of the *Journal of Pharmacy* gives the following directions for preparing a syrup of cubebs, which has been found an elegant as well as efficacious remedy in diseases of the throat and lungs: Fld. ext. cubebs, f℥ij.; carb. magnesia, f℥ss.; sugar, powdered, ℥xij.; orange-flower water, f℥ij.; water. q. s.; ess. oil almonds, gtt. j. Rub up the fld. ext. with the carb. magnesia, and then add ℥ij. of the sugar in small portions. When thoroughly mixed, add gradually first the orange-flower water, and then f℥vij. water, constantly triturating the mixture until the sugar is dissolved. Filter, and add q. s. water through the filter to measure f℥xj., in which dissolve the balance of the sugar without heat. Add the oil of almonds, put in a little alcohol, and again filter, adding, if necessary, q. s. water through the filter to measure one pint. The dose of this syrup is fʒj–iv., and it may be given in even larger doses, if desired.

Treatment of Erysipelas by the Application of Camphor in Ether.—Cover the parts affected from the first day, and during the continuance of the disease, with a strong solution of camphor in ether, composed of one part of camphor to two of ether. It is applied by means of lint wet five or six times a day, and then touching all the parts. The ether evaporates and leaves the surface covered with a light coat of camphor, which appears to possess great powers over the progress of the erysipelatous inflammation.

CHANGE OF LIFE IN HEALTH AND DISEASE.—The reviewer of Dr. Tilt's work on "Change of Life in Health and Disease," (*Am. Practitioner*), refers to the ganglionic system as a very common seat of those nervous affections which are incident to the change of life. In five hundred women treated by Dr. Tilt for various affections at this period of life, this system bore the onus of the disease in four hundred and six instances, in the greater number taking the form of epigastric faintness and sinking. The brain rarely escapes disorder in women who suffer at the change of life. In five hundred women treated by Dr. Tilt, nervous debility was encountered four hundred and fifty-nine times, headache in two hundred and eight, and varous other symptoms of brain disturbance five hundred and ninety-four times; or, in other words, five hundred women divided one thousand two hundred and sixty-one forms of cerebral disease. In his thirty-first table he gives the proportion of cases in which the gastro-intestinal organs were effected, and shows that they were in the proportion of three hundred and fifty-four to five hundred. The obstinacy of the biliary symptoms was the chief feature in many of the cases.

Dr. Tilt asserts that debility underlies chlorosis, palpipations and hysterical asthma. The remedies are sedatives, alkalies, diaphoretics, and, above all, tonics. He cannot sufficiently praise the utility of sedatives applied at the pit of the stomach. Belladonna or opium, or atropia very cautiously, or chloroform, may be rubbed over the epigastrium or used in the form of a plaster. Opium, hyoscyamus, laurel-water, or some other anodyne, is demanded internally, and respect must be constantly had to the state of the bowels.

MONS. LEFORT has made analyses of water taken from wells in the neighborhood of graveyards, and finds the water situated one hundred and fifty feet from graveyards to have a sweet, fetid taste, and upon evaporation by heat, a thick, grayish residue remained, which had a strong empyreumatic odor. Upon adding reagents, carbonic acid was given off, and salts of ammonia were thrown down. He thinks that no bodies of men or animals should be buried nearer than three hundred feet to any well from which water is taken for household purposes.

THE CONJOINT USE OF THE SULPHATES OF CINCHONA AND QUININE.—Sulphate of cinchona and sulphate of quinine are both extensively employed both in Europe and this country. When conjointly administered, it is claimed they assist each other and lessen the necessary quantity when used separately. Two parts of sulphate of cinchona to one of quinine are the proportions recommended. Take a hint.—*Medical Archives.*

Cauterization of the Urethra.—William Acton, M.R.C.S., (Functions and Disorders of the Reproductive Organs), thus performs cauterization of the urethra: He employs a syringe made of stout glass, and with it injects a ten-grain-to-the-ounce solution of nitrate of silver. The instrument is passed down the urethra, the patient standing against the wall. Within half an hour the patient may return home. Relapses do not often take place after this treatment, and he never employs any other plan. His instrument is obtainable at Fergusson's, Giltspur street, London. Tea, coffee and tobacco he looks upon as so many poisons for persons suffering from nervous depression from emissions. In his work, Mr. Acton quotes from a letter of the late Sir B. Bodie to a patient who consulted him, "You must not expect to be free from nocturnal emissions until you are married," without any comments.—*New York Medical Record.*

Alterative Syrup.—Professor E. Freeman, of Cincinnati, in the *Eclectic Medical Journal*, recommends the following as being an excellent alterative syrup:

℞.—Figwort root, lbs. 2; blue flag root, lbs. 1½; burdock root, tts. 1; bayberry root, tts. 1½; Butternut (inner part of root), tt. 1; mandrake root, tt. ¼; queen's root, tts. 1½; coriander seeds, ℥vj.; prickly ash berries, ℥vj.

Reduce the roots to a coarse powder and extract their strength by percolation with two and a half gallons diluted alcohol, and then, with boiling water, next evaporate to two and a half gallons, and add ten pounds of sugar. The coriander seeds and prickly ash berries are boiled in a small portion of the fluid before the sugar is added, strained, pressed and added to the syrup. Dose, from a teaspoonful to a tablespoonful three times daily.

Orchitis Treated with Absolute Rest.—Dr. Brambilla, of Lodi, Italy, publishes, in the *Lombard Medical Gazette*, an account of twenty-two cases of orchitis treated by this means; twelve of these were gonorrhœal, six traumatic, and four idiopathic. In all, the cure was complete. Medium time of treatment was five days, "although on admission the affection had lasted from two to six days, and the testis had acquired from two to five times its normal size. By absolute rest is meant that the patient should lie continuously on his back, with a small cushion between the thighs, in order to support the testis, and that he must never get off the bed even for the purpose of attention to his natural wants." The Doctor thinks orchitis can be more rapidly and more effectually cured in this way than by any other ordinary means.—*Chicago Medical Journal.*

Bromides in the Summer Complaints of Children.—Dr. F. G. Williams, of Watertown, Wisconsin, writes to the *Chicago Medical Examiner* that he has as much confidence in the use of bromide of potassium in the summer complaints of children as Dr. Caro, of New York, who has long advocated its use. At first Dr. W. used the bromide alone in simple solution, gr. xx. to gr. xxx. to ℥j. Now he is in the habit of using syrp. rhubarb as a menstruum. ℞. Syrp. rhubarb, ℥ij.; potass. brom., ℈ij. M. S. gtt. x. to xx. every one, two or three hours. If there is much pain or restlessness, from one-half to one grain morph. sulph. is added.

One of the most reliable stimulants is this: ℞. Hoffman's anodyn. paregoric, tinct. camphor, aa. ℥ij. Dose, from ʒj. to ʒiij., moderately diluted.

When the case proves rebellious, the vomiting and purging resisting the treatment just described, the following formula is a valuable auxiliary stimulant: ℞. Tinct. lavend. comp., tinct. cantharid., aa. ʒiij.; tinct. nuc. vomic. (alcohol), ʒijss.; diospyros virgin. (fruct. immat.), ʒiijss.; tinct. capsic., ℥iij.; tinct. opii (laudanum), ℥iss.; tinct. rhubarb. Dose, from half an ounce to one and a half, mixed with a small quantity of water.—*New York Medical Record.*

Delirium Tremens.—Dr. John Barclay (*London Lancet*) says: I was called late one night to see a young man who was laboring under this complaint. He had attempted to hang himself, had been rescued, and was then very violent. About half an hour before I saw him, he had got half a drachm of solution of morphia, which seemed to have no quieting effect whatever. I then injected hypodermically half a grain of morphia, and waited an hour; but at the end of that time he was still as violent as ever. I then injected half a grain more, and waited for two hours; but even then the violence was, if anything, greater than before. I now sent for a drachm of chloral in solution, half of which I gave him. In ten minutes he was fast asleep, and he slept for ten hours. When he awoke, he was slightly collected; he was well fed with strong soup and arrowroot, got the rest of his chloral draught, fell asleep again, and in twelve hours more awoke almost rational.

Impotence.—William Acton, M.R.C.S., in his work on "The Functions and Disorders of the Reproductive Organs in Childhood, Youth, &c.," speaks with some favor of cantharides and of phosphoric acid in the treatment of impotence. Strychnia, also, he considers to be a good tonic, and he much approves of electricity, occasionally, applied under the direction of a medical man. Bands worn promiscuously round the neck, etc., are worse than useless; they are often injurious.

Contagiousness of Purulent Secretions.—Dr. Hiller, says the London *Lancet*, in an inaugural dissertation on this subject, maintains the truth of the old view that all purulent secretions are contagious when applied to healthy mucous membrane. He states that pus taken from the vagina of a bitch in which inflammation had been excited by the injection of ammonia produced pyorrhœa in the urethra of a dog into which it was injected, though not before the latter had been mechanically irritated. If no mechanical irritation were employed, no catarrh occurred. The pyorrhœa, both in the male and the female animal, appeared to be closely analogous in its nature to gonorrhœa, since it spread in the former to the posterior part of the urethra, and in the latter to the mucous membrane of the uterus. M. Hiller holds that the same obtains in the case of the eye, and that all purulent discharges applied to the conjunctiva are capable of exciting inflammation in it. It is certain, however, that the matter from an inflamed lachrymal sac, or from an abscess of the lid, may freely enter the eye without producing a purulent discharge.

Nasal Hemorrhage.—There are few physicians who have not occasionally been annoyed by the difficulty with which nasal hemorrhage is arrested. An old shipmaster communicated to me a method, which shows that the artery furnishing the supply of blood can be perfectly compressed at the root of the incisor teeth. His process was to roll up a piece of paper and place it under the upper lip. The first opportunity I had of trying it was a case of profuse hemorrhage from a fall, which had persisted four days, notwithstanding repeated plugging of the nostrils, and the patient had become almost exsanguious. In this case the front teeth of the patient were wanting, and I applied the pressure by tying a knot in a bandage, which I placed *on* the upper lip so as to make pressure immediately at the root of the septum narium, and tied the bandage around the head above the ears. The hemorrhage was immediately and permanently arrested. On mentioning the subject to several of my medical friends, I found the practice was new to them all, and I therefore communicate it for the benefit of the profession.—*S. R. S.*

Nitrate of Silver in Jaundice and Chronic Gastritis. Dr. Peebles, of Petersburg, recommends in cases of jaundice the internal use of nitrate of silver, in doses of $\frac{1}{4}$ of a grain twice a day, on an empty stomach. Improvement is often observed on the second day, and ten days is the longest time required to remove the disease. The *modus operandi* is probably by correcting a state of irritation of the duodenum, on which the disease often depends.

Chorea.—Dr. A. J. Gupton, of New Providence, Tennessee, (*Nashville Journal of Medicine and Surgery*), reports a case of obstinate chorea in a woman, when four months *enciente*, who was relieved by the following treatment: ℞. Quevennes iron, gr. xxiv.; extr. hyoscyamus, gr. xij.; extr. cannab. indica, gr. vj. Ft. mass, and divide into twelve pills. S. One three times a day. This treatment was continued for thirty days, at the end of which she was entirely well. She went her full term, and was delivered of a sound, healthy child of six and a half pounds. Her general health kept good, with no symptoms of returning chorea until the next year, when, being again pregnant, of about the same length of time, she was suddenly seized with chorea again. He was firmly persuaded that the exciting cause of the first attack was the gravid uterus. Another coming on just at the same time in pregnancy, convinced him that his conjectures were correct. He therefore reasoned that the nerves which supply the uterus were excited by the growth of that organ, and, consequently, took an inordinate action, producing unusual activity in the sympathetic system, thus involving the brain and the whole nervous tissue, and chorea was the result.

He put her upon the treatment as before, with the addition of strychnia. On the fifteenth day of her treatment she went home nearly well. The treatment was ordered to be continued for thirty days longer.

Chloral and Strychnia.—M. Rabuteau, at the sitting of the Societe de Biologie (August 3), denied the alleged antagonism in the action of chloral and strychnine, asserted recently by M. Ore, which has been much discussed. His experiments lead him to opposite conclusions. Moreover, he considers that the experiments of M. Personne place it beyond doubt that chloral, administered in small doses, acts like chloroform administered in a continous manner. In a large dose, there is a transformation of a part of the chloral; the other acts by its own properties. We have, then, to do with a poison which would add its effects to those of the strychnine, if the latter have been previously injected.—*British Medical Journal.*

The Properties of Horse-Mint.—Dr. W. C. Buckley states that the oil of this variety of mint is a powerful rubefacient. During the prevalence of the epidemic typhus in Philadelphia, which occurred several years ago, Dr. E. A. Atlee was much pleased with the effects of the oil, applied in the form of a liniment, in the treatment of that malady. His formula was as follows: Oil of horse-mint, ℥ss.; tincture of camphor, ℥ij.; laudanum, ʒij. M. During the sinking stage, the arms, legs and breast were bathed, and, shortly, a comfortable glow succeeded.

Treatment of Diarrhœa in Infants.—Dr. Weiser objects to the usual modes of treating diarrhœa in children by means of stringent mixtures, warm poultices on the belly, and injections of mucilaginous fluids; instead, he prefers to envelop the child in a moist cloth, with a warm covering around this; when free perspiration has been induced, the skin is rubbed with a moist cloth. He gives a little coffee and milk with a small quantity of carbonate of soda several times daily. If the temperature of the body has been reduced by these means, cold cloths, changed every five or ten minutes for some time, are applied, and he prescribes five drops of the solution of acidulated sulphate of iron four times a day.—*Wien. Med. Wochenschrift.*

Aperients in Eczema.—Dr. J. L. Nilton, Surgeon to St. John's Hospital for Skin Diseases, (*Medical Press and Circular*), says that of all aperients in eczema, those containing magnesia seem to answer best. Henry's calcined magnesia is an excellent preparation; a teaspoonful taken in milk before breakfast will produce an action on the bowels which few other agents can effect. If the patient happens to be suffering from acidity, the beneficial action of the remedy is two-fold. The efficacy of calcined magnesia is, however, very decidedly increased by the addition of a drug which is as unpleasant as it is valuable—sulphate of magnesia.

Epilepsy Cured by the Use of Bromide of Potassium and Sulphate of Atropia.—Dr. L. P. Yandell, Jr., (*American Practitioner*), reports three cases of the above disease. In the first, a girl thirteen years of age, the bromide alone had been used in full doses for many months without relief. Dr. Y. gave her twenty-six grains of the bromide three times a day, and one hundredth of a grain of the sulphate of atropia night and morning. The attacks soon became less frequent, and in seven months had apparently disappeared. She was directed, however, to continue the medicine twelve months more.

Treatment of Tic-Douloureux by Ice.—In the *Mitt. des Aerztl. Vereins in Wien*, No. 7, 1872, (*Philadelphia Medical Times*), W. Wentteritz relates a very stubborn case of facial neuralgia in a lady, which had not yielded to any of the means applied. A smooth piece of ice was stroked gently over the affected side of the face every five minutes. The painfulness of the application was lessened by holding some alcoholic fluid in the mouth until a slight feeling of warmth was excited. The pain, which disappeared in twelve hours under this treatment, had not returned ten months subsequently.—*British Medical Journal.*

On the Employment of Nux Vomica and the Salts of Strychnine in the Treatment of Obstinate Vomiting.—The author of this communication, (Dr. Debange), relying upon the experiments of Magendie and Marshall Hall, which established the action of nux vomica upon the pneumogastric nerves, has tried strychnine in the treatment of asthma and emphysema with much success. These results have led Dr. Debange to employ the same medicinal agents in certain affections of the stomach, and especially in obstinate vomiting. He reports the case of a young woman, aged twenty years, who suffered from various nervous symptoms, among which figured obstinate vomiting, occurring after various hematemeses. This vomiting lasted for several days; blisters dressed with morphia had no effect. At last sulphate of strychnia, in doses of two milligrammes, was administered hypodermically. The success was immediate, and in the course of two days the vomiting was arrested. The administration of the strychnia was continued in the same doses for four days, and the cure was permanent. *Lyon Medical.*

Acute Sclerotitis and Iritis.—Dr. J. K. Spender, *Med. Chir. Rev.*, recommends the eye to be kept closed, and to give the patient one-twelfth of a grain of morphia every hour for the first twenty-four hours; during the next twenty-four or forty-eight hours, to increase the dose to one-eighth or one-tenth of a grain every two hours. The diet must be restricted, and an occasional purgative administered. During the treatment, a compress of lint dipped in hot water is to be kept on the eyes.

Catarrh Cut Short.—Dr. Dobell recommends the following plan for this purpose: Sesq. carb. of ammonia and morphia for two days, with ℥jss. liq. ammon. acet. the first night, and a comp. colocynth pill the second. In confirmed catarrh and bronchitis, in addition to medicine and hygienic regulations, winter residences in warm latitudes, and a summer sojourn in high localities, are recommended.

Nervous Sick Headache.—One-fourth of a grain of ipecac, repeated every half hour or hour, has relieved many cases of this affection, and if the ipecac is continued in one to three-grain doses, three or four times daily, a cure will frequently result—at least the intervals will be prolonged.

Removal of Stains of Nitrate of Silver.—Wet the linen in a solution of bichloride of mercury, one part to thirty-one, and rub it well, and then wash it in cold water.—*M. M.*

How to Take a Cold Bath.—Not every one, says a cotemporary, knows how to take a cold bath. It is a popular theory that the right thing to do is to jump sharply out of bed and to rapidly deluge the skin with showers of cold water, drying it with vigorous friction. This, however, is suitable only for the most hardy constitutions. The true way to take a tubbing in the morning is to rub the skin vigorously, using dry friction for at least five minutes before the bath, and not to bathe in cold water until the capillary circulation has been thoroughly stimulated. In this way it is well able to resist the shock; the lowering of the temperature, and the coldness and shivering, which sometimes follow the cold bath, are in this way avoided.—*Medical and Surgical Reporter.*

Expectorant Mixture.—The *Journal of Materia Medica* states that the following recipe is very effective in treatment of coughs, colds, asthmatic difficulties, etc.: ℞. Tinct. benzoin co., cong. iss.; tinct. sanguinariæ, tinct. lobeliæ, tinct. opii camph., syr. scillæ, aa. Oiii.; ol. helianth. ann., ℥v.; ol. sassafras, ℥ii.; mellis colat. opt., lbs. xii. M. Dose, a dessertspoonful every three or four hours, and more frequently if the cough is hard and expectoration difficult.

Pulmonary Consumption.—Dr. Thomas B. Peacock, of London, (*St. Thomas Hospital Reports*), in his excellent "Remarks on the Different Forms of Pulmonary Consumption," states that those cases do best which improve only slowly, and he has learned by experience to distrust a too rapid amendment, and that it is better in such cases to reduce the diet and other tonic measures.

Poisoning by Nitrate of Silver.—Dr. Ernest Hart, of London, (*British Medical Journal*), says that milk is an excellent antidote to nitrate of silver, in virtue of its large proportion of suspended albumen. He uses it in lieu of salt and water, for neutralizing the excessive effects of even the mitigated caustic, when employing it locally on the mucous membrane of the eyelids.

For Itching of the Anus.—Use the following ointment: Glycerin, ℥i.; purified tar, ʒss.; and with the aid of heat, powdered starch, ʒss. This makes an ointment of thin consistence, and easily spread. It dries up excoriations, checks exhalation, and dissipates slight cutaneous phlegmasiæ. Another preparation of pitch is the following: Cod-liver oil, two parts; oil of pitch, one part; used for itching and excoriations, as the other.

Locomotor Ataxia.—D. E. Dickerson, M.D., (*Kansas City Medical Journal*), relates two cases of this affection which were successfully treated with nitrate of silver.

Acute Traumatic Phlebitis.—J. L. Teed, M.D., Kansas City, Missouri, (*Kansas City Medical Journal*), reports the case of a young man, aged seventeen years, with acute traumatic phlebitis following severe contusions, treated successfully by carbonate of ammonia in large doses. This patient took thirty grains of carbonate of ammonia every two hours for sixty hours; then every four, for thirty-six hours; making, in all, nineteen drachms in ninety-six hours, together with three drachms of quinine. The dangerous symptoms were at once arrested, and he finally entirely recovered. The inflammation had passed up the deep veins, and was descending along the long saphenous vein.

Hydrate of Chloral in Pertussis.—The patient was a little girl, aged four, who was admitted on April 11, suffering from severe pertussis of about six weeks' duration, complicatad with bronchitis and pneumonia. Various remedies, including belladonna and ipecacuanha, produced little or no effect upon the paroxysms; but improvement at once followed the use of chloral in doses of five grains every four hours, and within a week the paroxysms had almost ceased.

Management of Scabies.—Dr. McCall Anderson (*Richardson's Hand-Book of Microscopy*) greatly prefers, in the treatment of scabies, in private practice, the following formula: ℞. Styracis liquidi, ℥j.; adipis, ℥ij. Melt and strain. This is a clean-looking ointment, has a pleasant aroma, kills the acari, and does not irritate the skin in the least, but, on the contrary, rather soothes it.

Carbolic Acid as a Local Anæsthetic.—Dr. C. Du Hadway, of Jerseyville, Illinois, (*Medical Archives*), has been using carbolic acid for the last two years as an anæsthetic in the removal of ingrowing toe-nails, also in abscesses, etc. He regards it as one of the best anæsthetics for these purposes, and uses the crystal in a state of deliquescence, full strength.

Dressing for Stumps.—Dr. George Buchanan, of the Glasgow Infirmary, advocates the following dressing for stumps: A few strips of lint dipped in carbolized oil—with a free opening for discharge to drain away—changed once or twice in twenty-four hours, according to the amount and nature of the discharge.

Diarrhœa.—The presence or absence of bile in the stools determines a very important point in the treatment. If the evacuations are bilious, opium is not only borne, but needed; if there is a deficiency of bile, opium will be injurious.

